# The formation of a rolling larval chamber as the unique structural gall of a new species of cynipid gall wasps

**DOI:** 10.1038/s41598-023-43641-6

**Published:** 2023-10-30

**Authors:** Tatsuya Ide, Asuka Koyama

**Affiliations:** 1 Department of Zoology, National Museum of Nature and Science, 4-1-1 Amakubo, Tsukuba, Ibaraki Japan; 2 Center for Biodiversity and Climate Change, Forestry and Forest Products Research Institute, 1 Matsunosato, Tsukuba, Ibaraki Japan

**Keywords:** Entomology, 3-D reconstruction, Biodiversity, Taxonomy

## Abstract

Insect galls, which often have complex external and internal structures, are believed to have adaptive significance for the survival of insects inside galls. A unique internal structure was discovered in the gall of a new cynipid species, *Belizinella volutum* Ide & Koyama, sp. nov., where the larval chamber could roll freely in the internal air space of the gall. Observations of the live galls using micro-computed tomography (micro-CT) revealed its formation process. The larval chamber becomes isolated from the internal parenchyma soon after the gall reaches the maximum diameter and is able to roll as the internal air space is expanding from the surrounding parenchyma to the outer gall wall. The enemy hypothesis could partly explain the adaptive significance of the unique structure of the gall of *B*. *volutum*.

## Introduction

Phytophagous insects have always seen their survival threatened by various natural enemies such as vertebrate and invertebrate predators and insect parasitoids^1,2^. Although their mortality by natural enemies accounts for a large proportion, behavioral and morphological adaptations against those enemies have been predicted in various of their life stages^3–5^. External body elements, such as caterpillar hairs and sclerotized beetle elytra, are some of the many examples of defensive structures in insects^6,7^.

**Figure 1:**
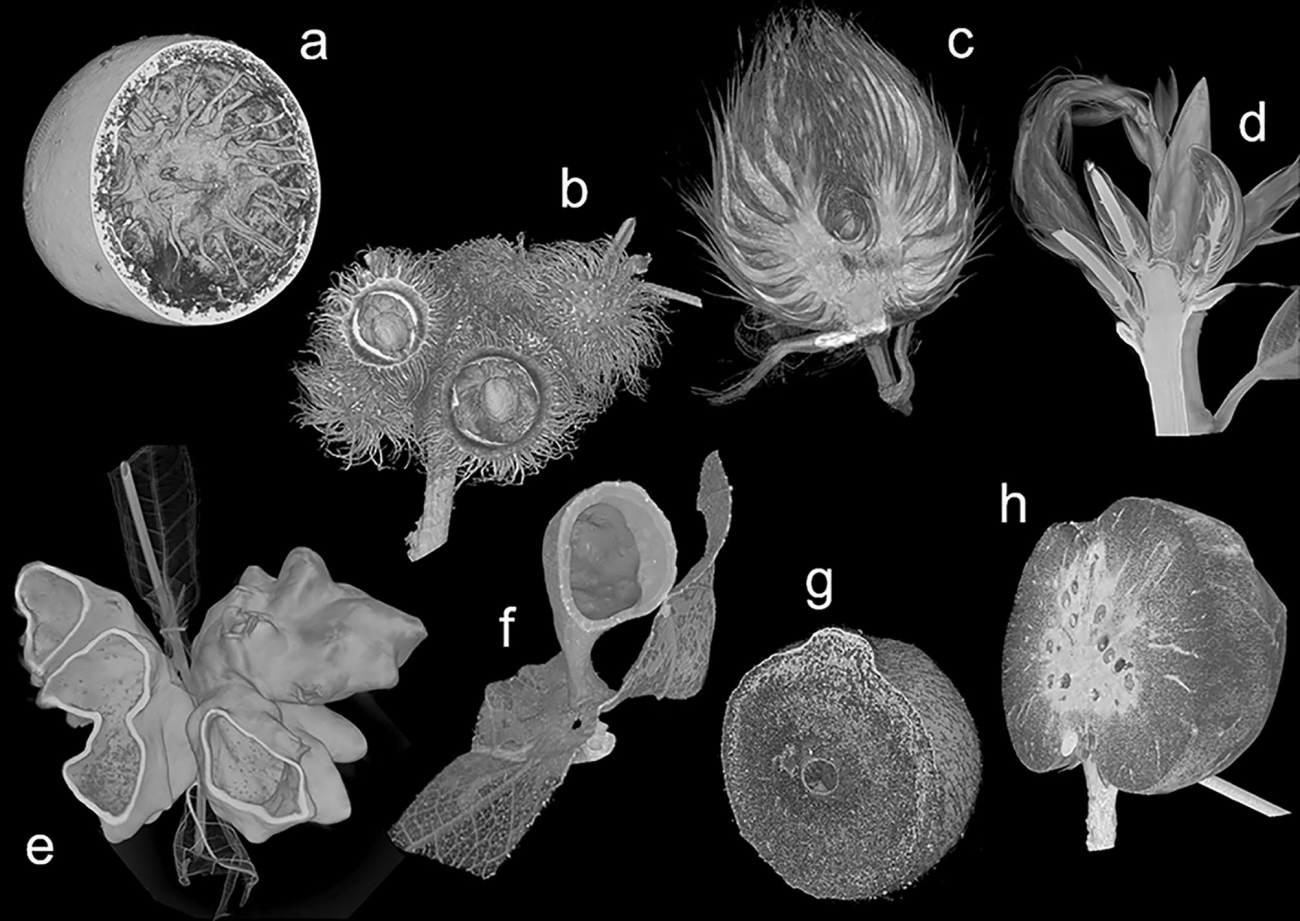
Diversity of external and internal structure of insect galls. (**a**) *Trichagalma acutissimae* (Monzen), asexual generation (Cynipidae). (**b**) *Trichagalma serratae* (Ashmead), asexual generation (Cynipidae). (**c**) *Andricus mukaigawae* (Mukaigawa), asexual generation (Cynipidae). (**d**) *Plagiotrochus masudai* Ide, Wachi & Abe, sexual generation (Cynipidae). (**e**) *Schlechtendalia chinensis* (Bell) (Aphididae). (**f**) *Paracolopha morrisoni* (Baker) (Aphididae). (**g**) *Cerroneuroterus japonicus* (Ashmead), asexual generation (Cynipidae). (**h**) *Biorhiza nawai* (Ashmead), sexual generation (Cynipidae). All images were visualized by micro-CT.

Plant galls induced by phytophagous insects are also believed to provide insects inside with some protection against predator or parasitoid attacks by various complex structures (Fig. 1)^8–14^. External gall structures such as dense coating of hairs or spines and hardness of gall wall may interfere with parasitoid or predator attack^12,13^, as occurs with caterpillar hairs and sclerotized beetle elytra. In addition, complex internal gall structures such as internal air space and the location and number of larval chambers may also interfere with their attack^12,13^. However, little has been experimentally confirmed that those complex structures contribute to the protection of insects inside.

Three-dimensional recognition of the complex structure of galls, as well as understanding their formation process, can contribute to a better understanding of their functionality. In this study, we report a unique internal structure discovered in a gall induced by a cynipid gall wasp (Hymenoptera: Cynipidae) on the oak *Quercus dentata* Thunb. and verify the detailed formation process of the structure through non-destructive and continuous observations using micro-computed tomography (micro-CT). Furthermore, we describe its gall inducer as a new species based on morphological and molecular identification. Finally, we discuss the adaptive significance of the gall structure of the new species based on the maturation process of the internal structure and the parasitoids observed.

## Results

### Structure of mature galls

**Figure 2:**
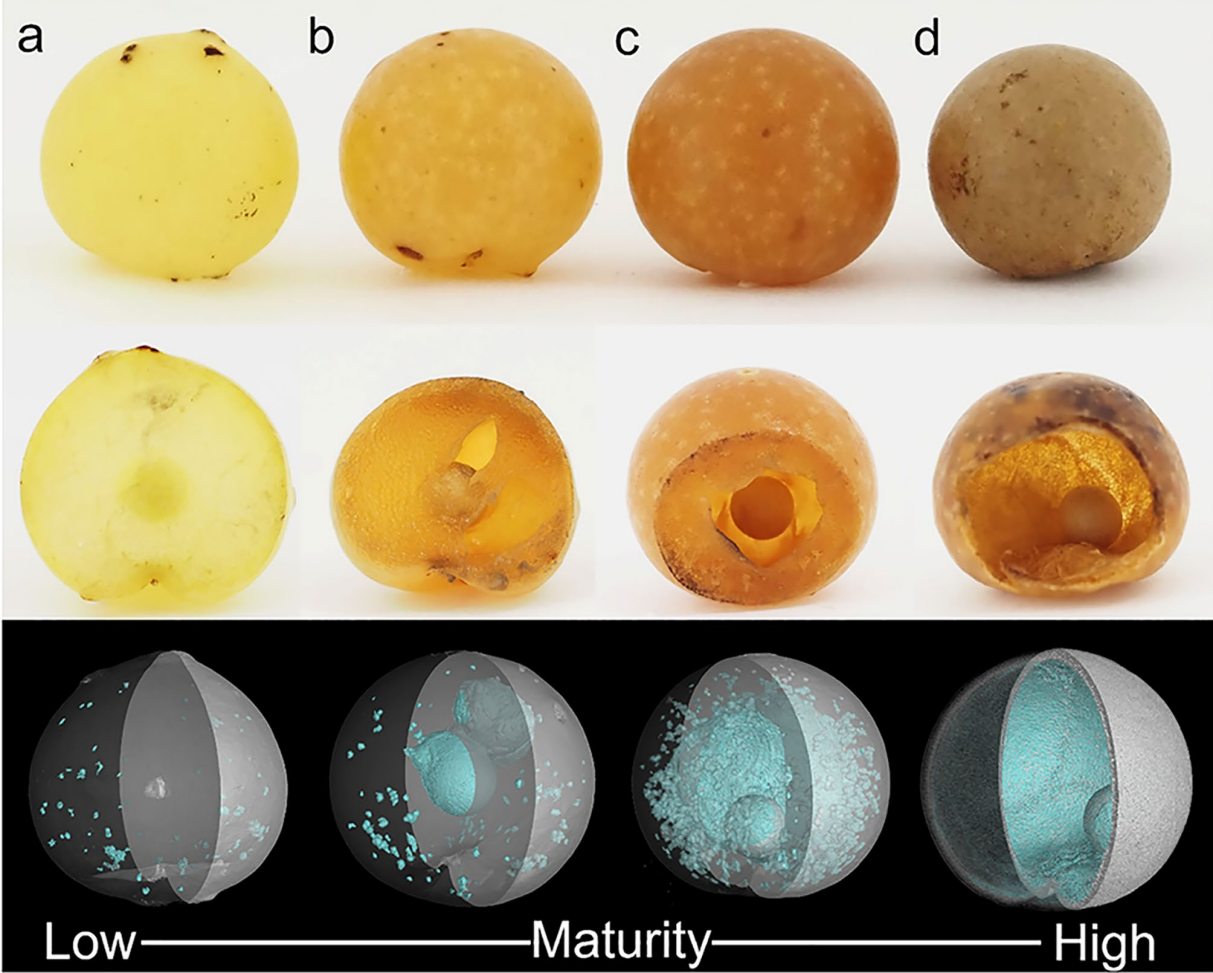
External and internal structure of *Belizinella volutum* galls with different maturity levels. (**a**) Low maturity. (**b**) Mid-low maturity. (**c**) Mid-high maturity. (**d**) High maturity. The top row shows the external structure, the middle row shows a longitudinal section by dissection and the bottom row shows the internal air spaces visualized by micro-CT (light blue).

**Figure 3:**
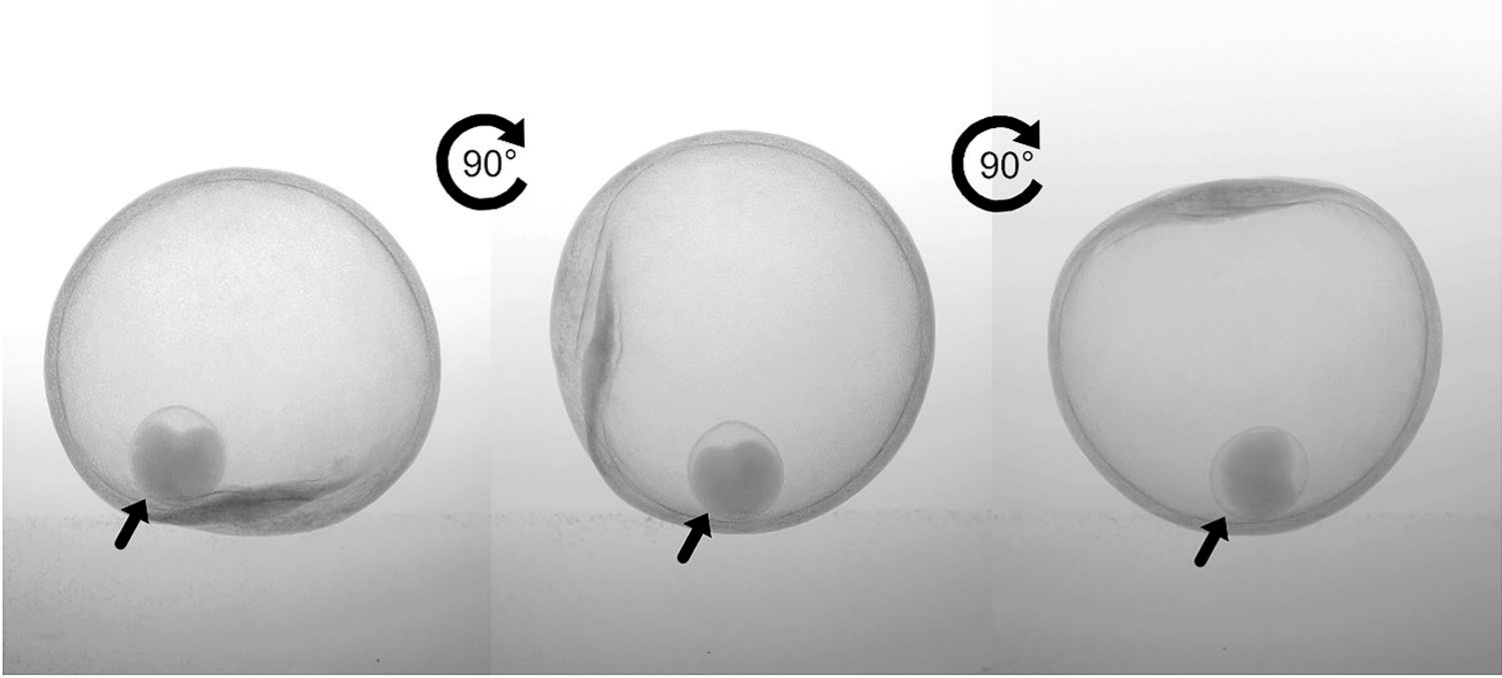
Movement of a larval chamber of mature *Belizinella volutum* gall at different angles visualized by micro-CT. The side of the gall contacting the leaf is at the bottom in the left column, to the left in the middle column and above in the right column. Each arrow indicates the larval chamber.

The galls of the new cynipid species are induced on the veins on the underside of *Q*. *dentata* leaves and usually do not detach after reaching maturity. Their maximum diameter is 10–12 mm. The external appearance is spherical, slightly depressed on the side adjacent to the leaf, yellow-green as they reach maximum diameter (Fig. 2a), then orange (Fig. 2b), and brown when mature (Fig. 2c). The outer wall is hardened and 0.46–0.99 mm thick. Most of the gall interior is hollow with a single larval chamber (Fig. 2d). The larval chamber is oval, 3.0 × 2.8 mm in diameter, milky white or light brown, and is not attached to the outer wall or internal parenchyma; therefore, it can roll freely and its position within the internal air space is indeterminate (Fig. 3).

### Temporal changes in the internal gall structure

**Figure 4:**
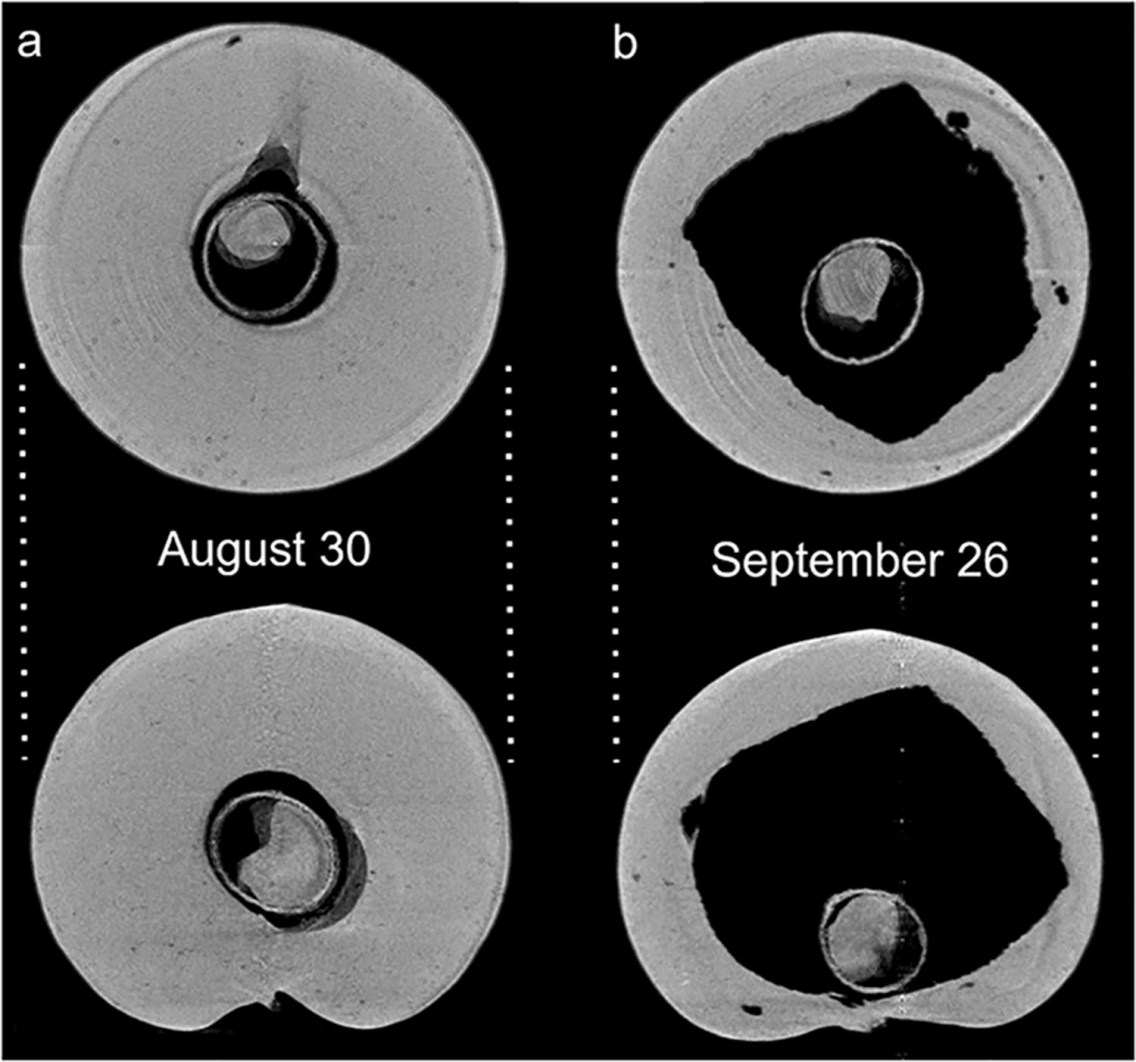
Temporal changes of internal structure of *Belizinella volutum* gall within a single specimen observed by micro-CT. (**a**) Internal structure on August 30. (**b**) Internal structure on September 26. The upper row shows transverse sections and the lower row shows longitudinal sections.

Temporal changes in the internal gall structure were observed within a single specimen by micro-CT (Fig. 4). The gall interiors of the four specimens at different maturity levels were also observed (Fig. 2, bottom). The gall interior was initially filled with juicy parenchyma without air spaces, and a larval chamber with a sclerotized wall was located in the center (Figs. 2a, 4a). An air space then formed between the larval chamber wall and the surrounding area of the parenchyma (Fig. 2b). A number of small air spaces arose in the parenchyma, and the central air space expanded in connection with them (Figs. 2c, 4b). Finally, a large air space accounted for most of the interior of the gall (Fig. 2d). As internal maturation progressed, the outer wall of the gall hardened (Supplementary Table S1).

### Higher taxonomy

Order Hymenoptera L., 1758; Superfamily Cynipoidea Latreille, 1802; Family Cynipidae Latreille, 1802; Tribe Cynipini Billberg, 1820.

### Genus *Belizinella* Kovalev, 1965

*Type species*. *Belizinella gibbera* Kovalev, 1965.

*Comments*. The genus *Belizinella* was synonymized with *Trigonaspis* in Melika and Abrahamson^15^ but was reestablished as an independent genus in Melika^16^. Only two eastern Palearctic species are known: *B*. *vicina* Kovalev and *B*. *gibbera* Kovalev^17,18^. *Belizinella* shares some diagnostic characteristics with *Trigonaspis* (e.g., the apterous female in the asexual generation, the arched mesosoma, and the shorter pronotum^15,19^) but is molecularly distant from it^16,20,21^.

### *Belizinella volutum* Ide & Koyama, sp. nov.

Cynipidae sp. 2: Koyama and Ide^22^: 4 (gall).

*LSID*:urn:lsid:zoobank.org:act:FD4F5E3B-CCED-4F7A-8B10-11EBC5E96ED4.

#### Holotype

**Figure 5:**
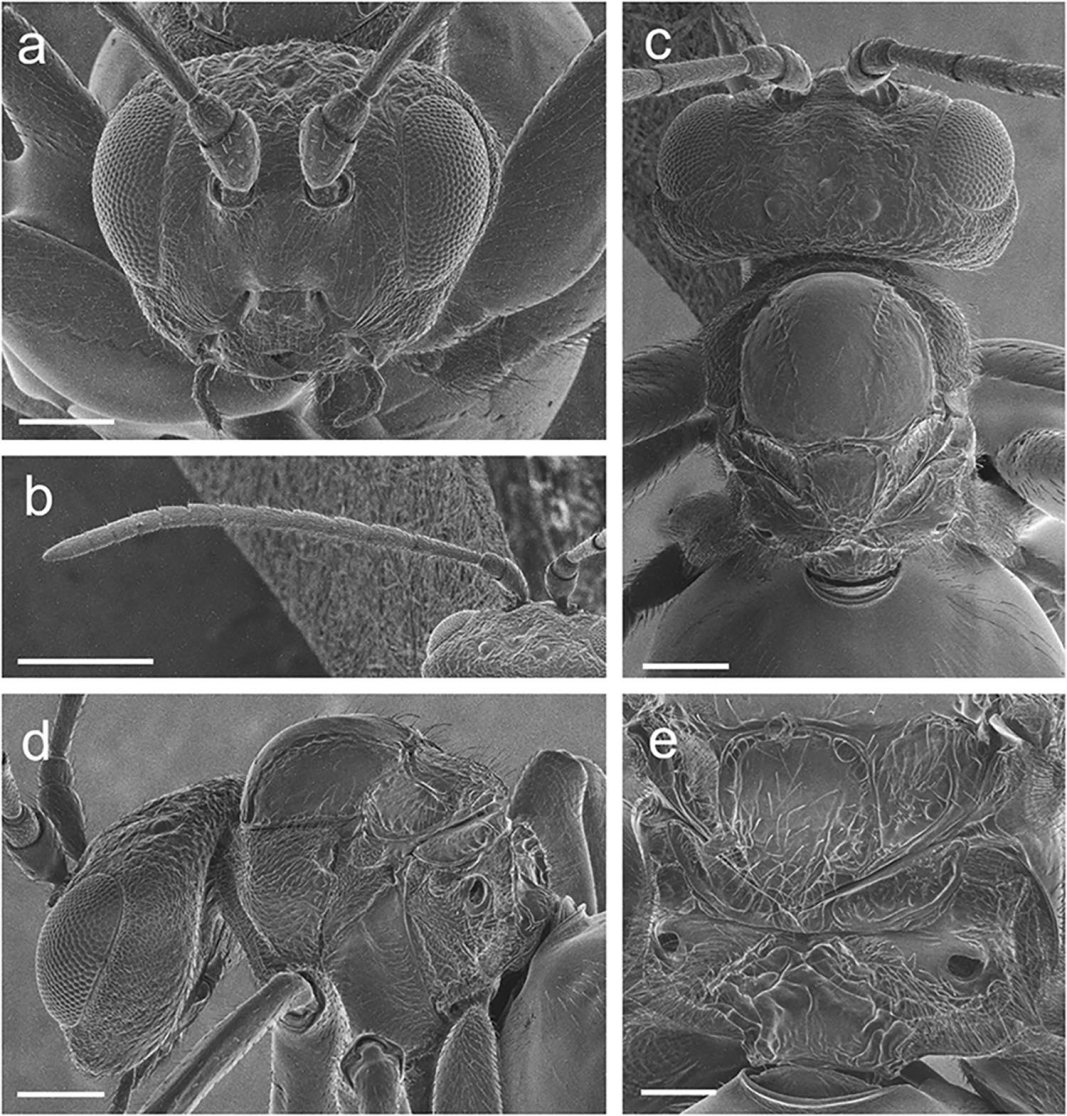
Head and mesosoma of *Belizinella volutum*. (**a**) Head, anterior view (scale bar 200 μm). (**b**) Antenna, dorsal view (scale bar 500 μm). (**c**) Head and mesosoma, dorsal view (scale bar 200 μm). (**d**) Head and mesosoma, lateral view (scale bar 200 μm). (**e**) Mesosoma, postero-dorsal view (scale bar 100 μm). All images were visualized by SEM.

**Figure 6:**
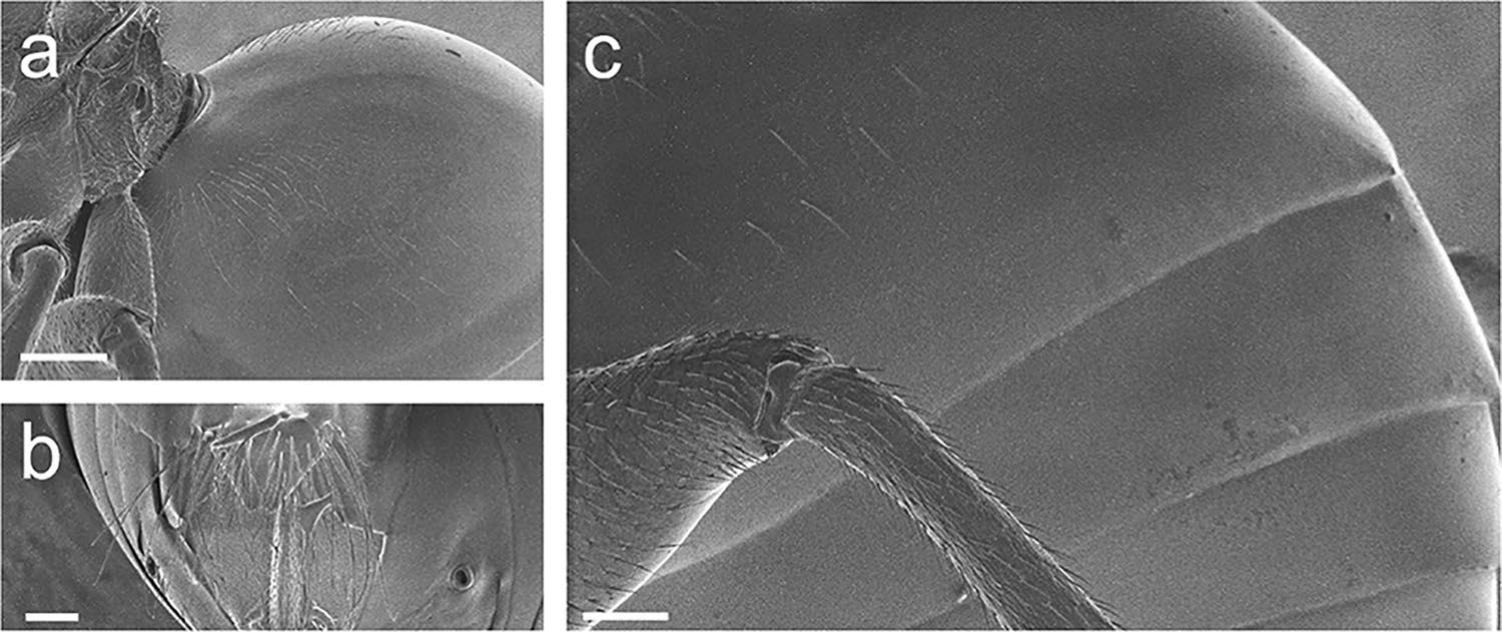
Metasoma of *Belizinella volutum*. (**a**) Anterior area of metasomal terga (scale bar 200 μm). (**b**) Hypopygial spine, ventral view (scale bar 100 μm). (**c**) Posterior area of metasomal terga (scale bar 100 μm). All images were visualized by SEM.

**Figure 7:**
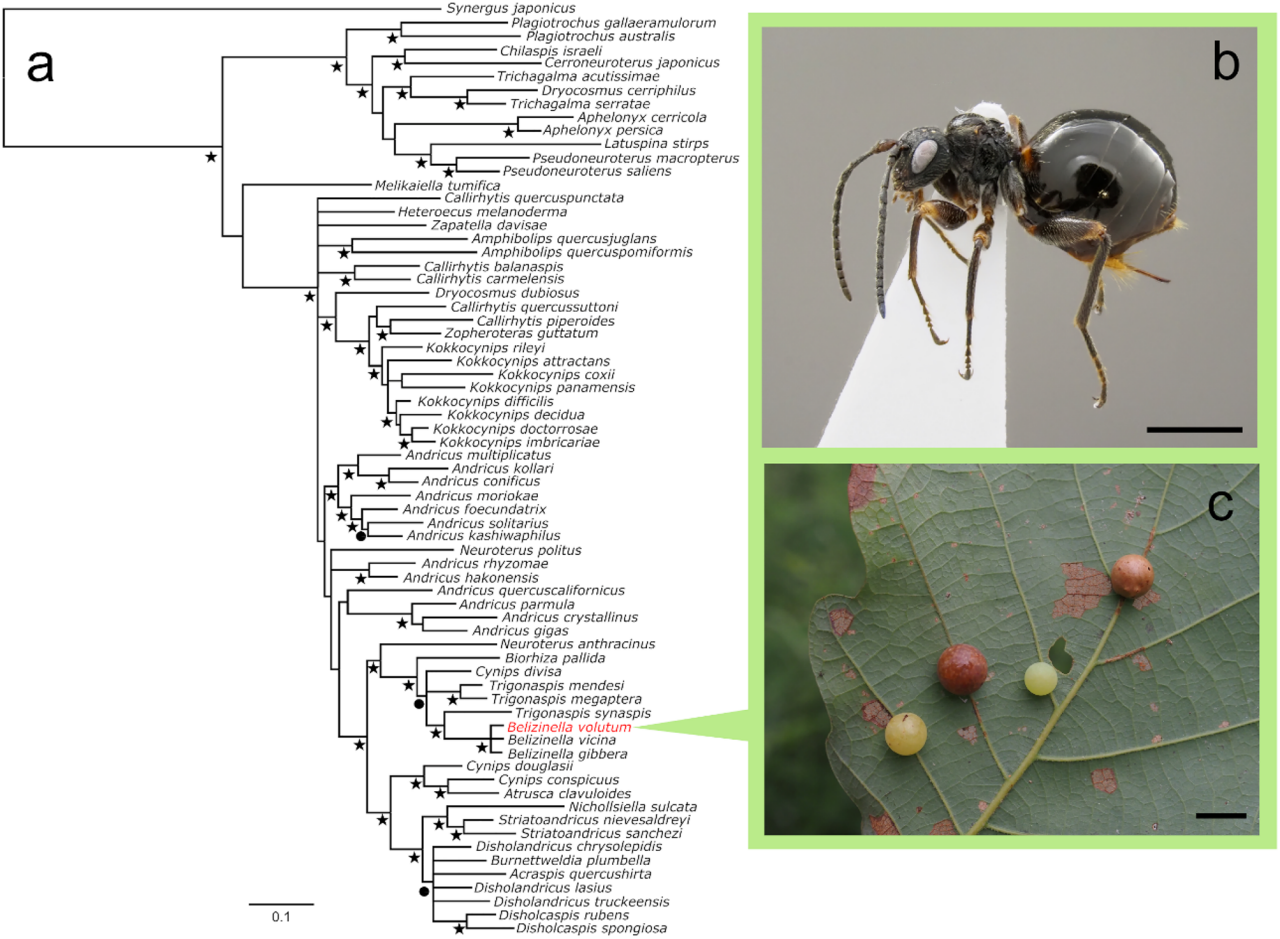
Phylogenetic position of *Belizinella volutum* within Cynipini. (**a**) Bayesian phylogenetic tree of species sampled from across the Cynipini, based on combined data of partial sequences of cytb and opsin regions. Black stars at nodes indicate ≥ 95% posterior probability support; black circles indicate 70–94% posterior probability support. (**b**) Habitus of *B*. *volutum* (holotype) (scale bar 1 mm). (**c**) Galls of *B*. *volutum* on *Quercus dentata* (scale bar 1 cm).

Female (Figs. 5, 6, 7b); JAPAN, Honshu, Okayama Prefecture, Maniwa, Hiruzen, 24. IX. 2021 (gall collection), 4. I. 2022 (adult emergence); host: *Q*. *dentata*; T. Ide and A. Koyama leg.; deposited at the Department of Zoology, National Museum of Nature and Science (NSMT), Tsukuba, Ibaraki, Japan (accession number: NSMT-I-Hym 77815).

#### Paratypes

Eleven females; same collection data as in holotype, except for adult emergence date: 1 female, 25. IX. 2021 (picked out from dissected gall); 1 female, 7. XII. 2021; 8 females, 24. XII. 2021; 1 female, 13. I. 2022. Four were deposited in the Smithsonian National Museum of Natural History (USNM), Washington, D.C., USA (accession numbers: USNMENT 01025516-01025519), and the remaining seven were deposited together with the holotype (accession numbers: NSMT-I-Hym 77816–77821).

#### Etymology

The new species is named after its unique internal gall structure: a free-rolling larval chamber. The Latin word "volutum" means “rolled”.

#### Japanese name

Insect name: kashiwa-suzu-tamabachi. Gall name: kashiwa-ha-suzu-tama-fushi. Each Japanese word means as follows: “kashiwa” = *Q*. *dentata*, “suzu” = small hollow Japanese bell that contains a pellet, “tamabachi” = cynipid wasps, “ha” = leaf, “tama” = ball, and “fushi” = gall.

#### Diagnosis

*Belizinella volutum* closely resembles *B*. *gibbera* but is morphologically distinguishable from the latter as in Table 1.

**Table 1:** Morphological diagnosis among three known *Belizinella* species.

	*B*. *volutum*	*B*. *gibbera*^23^	*B*. *vicina*^23^
Body length	2.5–3.3 mm	2.2–3.3 mm	1.9–2.8 mm
Tooth on tarsal claw	Absent	Present	Absent
Eye height/malar space	2.7	> 2.0	2.5
Ratio of distance from antennal rim to lower face margin to distance between antennal rims	2.6	2.0	–
Ratio of scape to pedicel	2.4	2.0	1.3
Ratio of F12 to F11	2.1	1.5	1.5
Setae on metasomal tergum II	Sparse	Sparse	Dense
Blunt process on lateral propodeal area	Present	Present	Absent

#### Description (holotype female)

Body length 2.88 mm. Head black, except for dark brown clypeus and lateral area of lower face. Mandible dark brown. Maxillary and labial palpi light brown. Antenna black, except for dark brown inner margin of scape. Mesosoma black. Legs dark brown except for black coxae and lighter basal and apical margins of femorae. Metasoma black except for lighter anterodorsal margin of metasomal tergite II, lateroventral area, and hypopygium.

Head 1.3 times as broad as high in anterior view, 2.1 times as broad as long in dorsal view, clearly broader than mesosoma in dorsal view (Fig. 5a,c). Mandible bidentate. Maxillary palpus 4-segmented; labial palpus 3-segmented. Oral foramen 0.9 times as broad as height of compound eye. Ventral clypeal margin incised medially. Anterior tentorial pit distinct. Epistomal and clypeo-pleurostomal sulci distinct. Lower face faintly coriarious, with sparse long setae; low longitudinal ridge extending medially from area between antennal rims to clypeus; facial strigae absent medially, but radiating from clypeus laterally, indicating subocular impression; distance from lower edge of antennal rim to ventral margin of lower face 2.6 times as long as distance between antennal rims. Malar space 0.4 times as long as eye height. Diameter of antennal rim almost as broad as distance between inner margins of rims and distance between lateral margin of antennal rim and mesal margin of compound eye. Transfacial distance 1.2 times as broad as height of compound eye. Direction of straight part of inner margins of compound eye slightly diverging ventrally. Gena finely coriarious, with sparse long setae, broadened behind eye, slightly visible in anterior view. Vertex and frons finely coriarious, with sparse short setae; POL 1.1 times as long as OOL, 1.8 times as long as LOL; OOL 2.0 times as long as distance from posterior edge of lateral ocellus to occipital margin in dorsal view. Occiput finely coriarious, with sparse short setae; occipital carina absent. Antenna with 14 antennomeres (Fig. 5b); relative lengths of scape, pedicel, and F1–F12: 17, 7, 20, 14, 12, 12, 10, 10, 9, 9, 8, 8, 7, 15.

Mesosoma higher than long in lateral view (Fig. 5d). Pronotum coriarious, with dense long setae; submarginal pronotal impression distinct, marked by ledge. Mesoscutum wider than long in dorsal view, concave in lateral view, faintly coriarious, with sparse long setae along notauli and lateral margin (Fig. 5c); impression mesad parascutal carina anteriorly continuing to anterior end of notaulus; notaulus superficial, indistinct posteriorly; anteroadmedian and parapsidal signa absent; median mesoscutal impression absent. Mesoscutellum rugose, with sparse long setae, flattened anteriorly, margined posteriorly and laterally; transscutal fissure distinct; scutellar fovea absent. Axillula delimited dorsally by ledge, with dense short setae; subaxillar bar narrow, not expanded posteriorly. Mesopleuron faintly coriarious, with dense long setae (Fig. 5d); femoral groove impressed from antero-lower margin of mesopleural triangle to postero-lower margin of mesopleuron, not sharply delimited; mesopleural triangle marked throughout by ventral ledge, with dense short setae. Metascutellum subrectangular (Fig. 5e); metanotal trough faintly rugose, with sparse short setae. Metepimeron distinctly impressed, with dense short setae (Fig. 5d). Propodeal spiracle margin raised and protruded; anterodorsal area near spiracle depressed. Lateral propodeal carina diverging ventrally, dorsally ending at level of margin (Fig. 5e); lateral propodeal area with blunt process postero-ventrally, with dense long setae; median propodeal area smooth, bare, with some irregular carinae. Anterior surface of mesocoxa protruding, peak close to base of coxa. Mesofemur 2.9 times as long as its widest part. Apex of metatarsal claw slightly bent, with sparse setae; base not expanded, without tooth. Length of metatibia 1.47 mm.

Metasoma smooth and polished, longer and broader than metasoma (Fig. 6a,c); metasomal tergite II extending more than half of metasoma, with patch of sparse setae antero-laterally; projecting part of hypopygial spine broad and short, 0.7 times as long as broad in ventral view, tapered to apex, with dense long subapical setae, extending far beyond apex of spine (Fig. 6b).

#### Variation

Lengths of body (mean ± S.D.) 2.52–3.33 (2.84 ± 0.20) mm, of metatibia 1.14–1.49 (1.35 ± 0.11) mm (n = 12). In some specimens, vertex and frons dull rugose, and anterior half of mesoscutellum coriarious.

#### Molecular profiles and phylogeny

Partial sequences of cytochrome c oxidase subunit I (COI), cytochrome b (cytb), long-wavelength opsin (opsin), and D2 loop of the 28S ribosomal RNA gene (D2) were determined in *B*. *volutum*. No species with highly matching sequences were identified in the database. The new species formed a well-supported clade with *B*. *vicina* and *B*. *gibbera* with 100% posterior probability support (Fig. 7a).

#### Biology

The gall is induced on the vein on the underside of the leaf (Fig. 7c), reaches its maximum diameter in August, and remains on the leaf after maturation. The pupa is observed in the gall before the end of September. The adult female emerges from the gall between December and January. Although the egg-laying site has not yet been confirmed, the egg is probably laid in the latent or winter buds, considering the developmental state of the host plant during the adult emergence season. The timing of gall formation and the morphological characteristics of the adults indicate that the above females are the asexual generation. Neither the sexual generation galls nor adults are known.

#### Parasitoid and inquiline

Of the 30 galls we collected in September 24, 2021, two of them yielded only *Torymus* sp. (Hymenoptera: Torymidae) each, 19 yielded only *B*. *volutum*, and 9 yielded no adults. Each of the two *Torymus* sp. emerged in July and September, 2022, under laboratory conditions. No inquilines emerged. The partial sequence of the COI gene was determined for *Torymus* sp. and deposited in GenBank under accession number OR339872. No species with highly matching COI sequences were identified in the database. Voucher specimens were deposited in the NSMT (accession numbers: NSMT-I-Hym 77822–77823).

## Discussion

### Free-rolling larval chamber formation

Cynipid galls can be divided into the larval chamber and the outer gall^11^. A simple-structured gall consists only of a larval chamber (Fig. 1d). When the outer gall is present, the larval chamber is surrounded by woody sclerenchyma or spongy parenchyma (Fig. 1a,b,c,g,h)^11^. In more complex galls, the larval chamber can be separated from the outer gall by complex internal air spaces (Fig. 1a,b). In the asexual generation galls of the two previously known *Belizinella* species, *B*. *vicina* and *B*. *gibbera*, their larval chambers are surrounded by parenchyma without any air space^23^. However, in the mature gall of *B*. *volutum*, the larval chamber is completely separated from the outer gall, and the position of the larval chamber within the gall is indeterminate. Although such unique internal structures with a free-rolling larval chamber have been reported in a few cynipid galls^12,24,25^, little is known about their formation process. This study visualized this phenomenon using micro-CT for the first time.

Cynipid galls develop in three phases: initiation, growth, and maturation^10,12^. Formation of the internal air space occurs during the maturation phase^10,12^; therefore, the formation of the free-rolling larval chamber progresses during the maturation phase. The gall interior of *B*. *volutum* is initially filled with juicy parenchyma without air spaces, as is typical of other galls of *Belizinella*. However, air spaces appear from the surrounding area of the larval chamber, and eventually, a large portion of the gall interior becomes hollow. Because the larval chamber and surrounding parenchyma are completely separated in the early stages of the mature phase, air space formation may be predestined for the growth phase. Internal air spaces are also observed in some fruits and seeds (e.g., fruits of *Sapindius* spp.^26^). Although gall formation is thought to involve the expression of a group of genes related to flower and fruit formation^27^, it remains unclear which group of genes is expressed during complex gall formation in gall wasps^28^. It is an interesting future challenge to determine the gene expression profiles in cynipid gall formation, including the free-rolling larval chamber of the gall of *B*. *volutum*.

### Adaptive significance of the free-rolling larval chamber

Nutrition, microenvironment, and enemy hypotheses have generally been proposed to account for the adaptive significance of insect galls^8,12,13^. Since the complex external and internal structures of insect galls are thought to have been acquired through interactions between cynipids and their natural enemies, parasitoids and predators^13^, the enemy hypothesis could account for the evolution of the complex structures of insect galls. Gall toughness, gall wall thickness, the number of larval chambers per gall, external hairs or spines, and internal air spaces are thought to be protective structures against natural enemies^12^. However, most insect galls are not free from natural enemies, even though they have complex internal structures such as free-rolling larval chambers^13,29^. Zhang et al.^29^ reported that a cynipid gall with a free-rolling larval chamber was attacked by a single species of *Sycophila* (Hymenoptrea: Eurytomidae). In addition, we identified a parasitoid, *Torymus* sp., that emerged from the galls of *B*. *volutum*.

The genus *Torymus* Dalman is one of the ectoprasitoids of Cynipidae and Cecidomyiidae and has a long ovipositor that penetrates the outer gall into the larval chamber and parasitizes the larvae inside the gall^30,31^. Although gall toughness and gall wall thickness are generally expected to contribute to protecting larvae inside, their protective effects might be obscured against such parasitoids. However, in the case of the mature gall of *B*. *volutum*, it is assumed that even if the tip of the ovipositor penetrates the outer gall wall and reaches the larval chamber wall, it cannot easily reach the inside of the chamber because the larval chamber is rolled within the gall when the ovipositor forces it to penetrate (Supplementary Video S1). Therefore, the *Torymus* sp. probably attacks *B*. *volutum* before the internal air space of the gall is completely developed. In fact, one of the two individuals of *Torymus* sp. emerged from the gall in July, when most of the galls of *B*. *volutum* did not reach their maximum diameter.

In addition to insect parasitoids, galling insects are under selective pressure from predators, such as birds^32^. Although further experimental studies are required, the unique structure of the gall of *B*. *volutum* might be advantageous for avoiding bird predation. Birds can easily locate the exposed chamber if the larval chamber is attached to the outer gall, whereas the larval chamber of *B*. *volutum* may roll away through a broken gall wall when birds vigorously break it. Although we did not directly observe bird predation on the galls of *B*. *volutum*, some broken and hollowed galls remained on the plants.

It is difficult to account for the adaptive significance of the free-rolling larval chamber of the gall of *B*. *volutum* by nutrition and microenvironmental hypotheses. The known habitat of *B*. *volutum* is restricted to abandoned semi-natural grasslands that had been managed by regular burning^22^. In addition, the grassland area is covered with snow for approximately three months during winter. Although the internal air spaces of the galls may mitigate rapid temperature increases^33^, it is still unclear whether the free-rolling larval chamber of the gall of *B*. *volutum* has advantages for their survival in such habitats.

### Continuous observation of the internal structure of live galls using micro-CT

We applied micro-CT to observe the internal structure of insect galls with live larvae. Micro-CT has recently been widely used to analyze the internal and external insect structures^34–36^. The main advantage of this method is the non-destructive and three-dimensional observation of the internal structure. Live plants are capable of continuous micro-CT in a living state^34^. It has also been shown that the continuous observation of live insects using micro-CT is possible^37,38^. In the present study, we observed the maturation phase of the cynipid gall after detachment from the plant; however, it may be possible to monitor gall development continuously from the initiation and growth phases using potted live plants. Non-destructive visualization of the internal structure of insect galls by micro-CT would contribute to future studies on understanding the adaptive significance of insect galls.

## Methods

### Collection and rearing of gall wasps

The type locality of *B*. *volutum* (Hiruzen, Okayama) is located in western Japan: 35° 17′ 14" N, 133° 35′ 41" E, with an elevation of approximately 600 m. It is a cool highland region, with average temperatures reaching 23 °C in the summer. Mountainous and forested areas are covered with snow between December and March. Permission is not required for insect gall collection at the study site.

Thirty galls of *B*. *volutum*, which were determined to have almost reached their maximum diameter, were collected from the field on the leaves of *Quercus dentata* in September 24, 2021. The collected galls were stored in moistened moss in square plastic cases with mesh lids. Those cases were kept in an incubator under the following temperature settings: 1 °C at 00:00–05:00, 5 °C at 05:00–19:00, 3 °C at 19:00–24:00 in January; 5 °C at 00:00–07:00, 10 °C at 07:00–09:00, 15 °C at 09:00–17:00, 10 °C at 17:00–24:00 in February and December; 10 °C at 00:00–05:00, 15 °C at 05:00–08:00, 20 °C at 08:00–19:00, 15 °C at 19:00–24:00 in March–May, September–November; 15 °C at 00:00–05:00, 20 °C at 05:00–08:00, 25 °C at 08:00–19:00, 20 °C at 19:00–24:00 in June–August. Galls were observed every 3–5 days. When the moss dried, water was added to keep it moist. Adult insects that emerged from galls were immersed in 99.5% ethanol.

### Examination of gall structures

Several additional galls were collected from the field in August 17, 2022, for micro-CT scanning. It was conducted using an inspeXio SMX-225CT FPD HR Plus (Shimadzu, Kyoto, Japan) with a tube voltage of 115 kV, tube current of 70 µA, and a slice thickness of 49 µm. Visualization and measurement of the micro-CT images were conducted using VGSTUDIO MAX ver. 3.4 (Volume Graphics KK, Aichi, Japan).

The observations and measurements of galls were performed in two ways. First, changes in the internal structure within a single gall specimen were observed at one-month intervals (August 30, 2022 and September 26, 2022) using micro-CT. Two gall specimens with different initial maturities were used in this observation. Second, four gall specimens with different maturities were selected and observed non-destructively using micro-CT in August 30, 2022. Then, longitudinal sections of the four galls were observed directly by dissection and were photographed. As an indicator of gall toughness, the force required to penetrate the gall wall was measured before dissection using a digital force gauge (ZTS-500N, Imada, Aichi, Japan) with Shiga insect pin No. 3 (Shiga Konchu Fukyusha, Tokyo, Japan) attached vertically to the tip of the instrument. The maximum force at the penetration point through the outer gall wall was recorded for all four gall specimens. Measurements were conducted using a pointed needle tip and a non-pointed needle head.

### Examination of adult morphology

Nineteen adults of *B*. *volutum* were yielded from the galls we collected in September 24, 2021. Adults were preserved in 99.5% (v/v) ethanol. Twelve adults of *B*. *volutum* were air-dried and mounted on the tips of triangular papers for morphological examination, and the remaining seven adults were preserved for future studies. Adult morphology was observed and measured using a stereomicroscope (S8-APO, Leica K.K., Tokyo, Japan) and a scanning electron microscope (JSM-6380LV, JEOL, Tokyo, Japan) operating at 1.5 kV. The lengths of each body part were measured using an ocular micrometer. Focus stacking was conducted for a photo of the adult habitus using CombineZP software (https:// combinezp.software.informer.com/). All images were processed into figure plates using GNU Image Manipulation Program (GIMP 2.10.20; https://www.gimp.org/).

The terminology used for morphological characteristics followed those of Ronquist and Nordlander^39^, Melika^19^ and Liljeblad et al.^20^. The terminology used for the surface sculpture followed that of Harris^40^. The following morphological abbreviations were used: POL, postocellar line (the distance between the inner edges of the two lateral ocelli in dorsal view); OOL, ocular-ocellar line (the distance from the outer edge of the lateral ocellus to the compound eye in dorsal view); LOL, lateral-ocellar line (the distance between the median and lateral ocelli in dorsal view); and F1–F12, the first to twelfth flagellomeres.

### DNA analysis

DNA was extracted from a single specimen of *B*. *volutum* adult using DNeasy Blood and Tissue Kit (Qiagen K.K., Tokyo, Japan). Partial sequences of COI, cytb, opsin, and D2 were amplified by polymerase chain reaction (PCR) using GoTaq Green Master Mix (Promega K.K., Tokyo, Japan). The following primers were used: LCO1490 (5′-GGTCAACAAATCATAAAGATATTGG-3′) and HCO2198 (5′-TAAACTCAGGGTGACCAAAAAATCA-3′) for COI^41^; CB1 (5′-TATGTACTACCATGAGGACAAATATC-3′) and CP2 (5′-CTAATGCAATAACTCCTCC-3′) for cytb^42,43^; D2B (5′-GTCGGGTTGCTTGAGAGTGC-3′) and D3A-r (5′-TCCGTGTTTCAAGACGGGTC-3′) for D2^44^; opsinGWF (5′-CTCCYTDTTCGGATGTGBHTCCATT-3′) and opsinGWR (5′-CCTTRGCRAGYTTATGTTCRG-3′) for opsin^45^. PCR products were purified using a QIAquick PCR Purification Kit (Qiagen K.K., Tokyo, Japan). Sequencing was performed by Eurofins Genomics K.K. (Tokyo, Japan) using a 3730xl DNA Analyzer (Thermo Fisher Scientific K.K., Tokyo, Japan). The complementary forward and reverse sequences were assembled using MEGA 7.0.26^46^. We also determined the partial sequences of each region for three species of the Japanese Cynipini: *Cerroneuroterus japonicus* (Ashmead), *Latuspina stirps* (Monzen), and *Trichagalma acutissimae* (Monzen).

Combined datasets of the cytb and opsin regions were created, including the aforementioned species, *B*. *vicina*, *B*. *gibbera* and other global Cynipini species presented by Fang et al.^21^ and Nieves-Aldrey et al.^47^ (Supplementary Table S2). We did not include the COI and D2 genes in our datasets because there were no available sequences for these genes in *B*. *vicina* and *B*. *gibbera*. The datasets were divided into seven partitions (the 1st, 2nd, and 3rd codon positions of cytb; the 1st, 2nd, and 3rd codon positions of opsin; and the intronic section of opsin). The best-fit model was selected for each partition, according to Nieves-Aldrey et al.^47^. Bayesian phylogenetic inference was carried out using MrBayes 3.3.3^48^ under the HKY + I + G model for partitions of the 1st and 2nd codon position of cytb, the HKY + G model for partitions of the 3rd codon position of cytb and opsin, and the HKY + I model for partitions of the 2nd and 3rd codon position and the intron of opsin. Posterior probabilities were estimated from MCMC runs of 10 million generations sampled every 1000 generations, with the first 25% of the samples discarded as burn-in in MrBayes. *Synergus japonicus* Walker was used as the outgroup, as in Nieves-Aldrey et al.^47^. The resulting phylogenetic tree was visualized using FigTree v1.4.0 (http://tree.bio.ed.ac.uk/software/figtree/) and Inkscape v1.2.2 (https://inkscape.org/).

### Nomenclature

This paper and its nomenclatural act have been registered in Zoobank (http://www.zoobank.org), the official registry of the International Commission on Zoological Nomenclature. The Life Science Identifier (LSID) number of this publication is: urn:lsid:zoobank.org:pub:9637286F-9F27-4663-9429-31804D9F2045.

### Statement

All methods in this study were carried out in accordance with the relevant institutional guidelines and regulations. No plants or leaves were collected during the study period.

### Supplementary Information

Supplementary Table 1.

Supplementary Table 2.

Supplementary Video 1.

## Data Availability

All new sequences were deposited in GenBank (https://www.ncbi.nlm.nih.gov/genbank) with accession numbers OQ745675–OQ745682, OQ754182–OQ754185, OQ754420–OQ754423, and OR339872.
