# Supplementary material for: The formation of a rolling larval chamber as the unique structural gall of a new species of cynipid gall wasps
Source: Sci Rep. 2023 Oct 30;13:18149. doi: 10.1038/s41598-023-43641-6 (PMC10616116; doi:10.1038/s41598-023-43641-6)
Supplement: Supplementary file 1 — Supplementary Table 1. [file 41598_2023_43641_MOESM1_ESM.docx]

**Supplementary Table S1**. Force to penetrate the outer gall wall of four *Belizinella volutum* gall specimens of different maturity levels (1 N = 1 kg ‧ m/s^2^).

|  | Maturity levels | | | |
| --- | --- | --- | --- | --- |
|  | Low (Fig. 2a) | Mid-low (Fig. 2b) | Mid-high (Fig. 2c) | High (Fig. 2d) |
| Pointed needle tip penetration | 0.3 N | 0.3 N | 0.5 N | 1.4 N |
| Non-pointed needle head penetration | 2.4 N | 2.9 N | 3.2 N | 4.5 N |
