# Supplementary material for: The formation of a rolling larval chamber as the unique structural gall of a new species of cynipid gall wasps
Source: Sci Rep. 2023 Oct 30;13:18149. doi: 10.1038/s41598-023-43641-6 (PMC10616116; doi:10.1038/s41598-023-43641-6)
Supplement: Supplementary file 2 — Supplementary Table 2. [file 41598_2023_43641_MOESM2_ESM.docx]

**Supplementary Table S2.** Cynipid species and the accession number of each sequence used for our phylogenetic analysis.

| Species | cytb | opsin |
| --- | --- | --- |
| *Acraspis quercushirta* | KX683594 | KX683479 |
| *Amphibolips quercusjuglans* | KX683595 | KX683480 |
| *Amphibolips quercuspomiformis* | KX683618 | KX683505 |
| *Andricus conificus* | AJ228460 | AF481719 |
| *Andricus crystallinus* | KX683596 | KX683481 |
| *Andricus foecundatrix* | AJ228452 | AF481725 |
| *Andricus gigas* | KX683598 | KX683483 |
| *Andricus hakonensis* | KX683609 | KX683495 |
| *Andricus kashiwaphilus* | KX683600 | KX683486 |
| *Andricus kollari* | EF031412 | AF481716 |
| *Andricus moriokae* | KX683602 | KX683488 |
| *Andricus multiplicatus* | DQ217996 | DQ217970 |
| *Andricus parmula* | KX683603 | KX683489 |
| *Andricus quercuscalifornicus* | KX683605 | KX683491 |
| *Andricus rhyzomae* | KX683607 | KX683493 |
| *Andricus solitarius* | AJ228475 | AF481724 |
| *Andricus truckeensis* | KX683610 | KX683496 |
| *Aphelonyx cerricola* | DQ218003 | DQ217972 |
| *Aphelonyx persica* | DQ218004 | KX683499 |
| *Atrusca clavuloides* | KX683627 | KX683516 |
| *Belizinella gibbera* | MT361688 | MT361722 |
| *Belizinella vicina* | MT361689 | MT361723 |
| *Belizinella volutum* | OQ745678 | OQ745682 |
| *Biorhiza pallida* | AJ228481 | AY371065 |
| *Callirhytis balanaspis* | KX683615 | KX683502 |
| *Callirhytis carmelensis* | KX683616 | KX683503 |
| *Callirhytis piperoides* | MW326677 | MW326715 |
| *Callirhytis quercuspunctata* | KX683619 | KX683506 |
| *Callirhytis quercussuttoni* | KX683620 | KX683507 |
| *Cerroneuroterus japonicus* | OQ745675 | OQ745679 |
| *Chilaspis israeli* | KX683622 | KX683509 |
| *Cynips conspicuus* | KX683613 | KX683500 |
| *Cynips divisa* | KX683623 | KX683510 |
| *Cynips douglasii* | KX683611 | KX683497 |
| *Disholcaspis chrysolepidis* | KX683590 | KX683475 |
| *Disholcaspis lasius* | KX683601 | KX683487 |
| *Disholcaspis plumbella* | KX683592 | KX683477 |
| *Disholcaspis rubens* | KX683569 | KX683837 |
| *Disholcaspis spongiosa* | KX683587 | KX683856 |
| *Disholcaspis sulcata* | KX683593 | KX683478 |
| *Dryocosmus cerriphilus* | DQ286808 | DQ217982 |
| *Dryocosmus dubiosus* | MW326678 | MW326716 |
| *Heteroecus melanoderma* | KX683624 | KX683512 |
| *Kokkocynips attractans* | KX683621 | KX683508 |
| *Kokkocynips coxii* | MW326680 | MW326717 |
| *Kokkocynips deciduus* | MW326683 | MW326718 |
| *Kokkocynips difficilis* | MW326684 | MW326719 |
| *Kokkocynips doctorrosae* | MW326687 | MW326720 |
| *Kokkocynips imbricariae* | MG821069 | MW326721 |
| *Kokkocynips panamensis* | MW326691 | MW326722 |
| *Kokkocynips rileyi* | MW326693 | MW326723 |
| *Latuspina stirps* | OQ745677 | OQ745681 |
| *Melikaiella tumifica* | MW326694 | MW326724 |
| *Neuroterus anthracinus* | DQ218015 | DQ217947 |
| *Neuroterus politus* | DQ218016 | DQ217949 |
| *Plagiotrochus australis* | DQ218026 | DQ217979 |
| *Plagiotrochus gallaeramulorum* | DQ218023 | DQ217946 |
| *Pseudoneuroterus macropterus* | AF539588 | DQ217953 |
| *Pseudoneuroterus saliens* | AF539589 | KX683514 |
| *Striatoandricus nievesaldreyi* | MW326695 | MW326725 |
| *Striatoandricus sanchezi* | MW326696 | MW326726 |
| *Trichagalma acutissimae* | OQ745676 | OQ745680 |
| *Trichagalma serratae* | KX683626 | KX683515 |
| *Trigonaspis megaptera* | MT361701 | MT361736 |
| *Trigonaspis mendesi* | MT361702 | MT361737 |
| *Trigonaspis synaspis* | AF539591 | DQ217956 |
| *Zapatella davisae* | MW326697 | MW326727 |
| *Zopheroteras guttatum* | MW326698 | MW326728 |
| *Synergus japonicus* | MW326699 | MW326729 |
